# Effect of Thermoactivated Recycled Cement, Hardened Cement Powder and Hydrated Lime on the Compressive Strength of Mortars

**DOI:** 10.3390/ma17164002

**Published:** 2024-08-12

**Authors:** Hassan Fardoun, Guilherme Ascensão, Pedro Mantas, Victor Ferreira

**Affiliations:** 1 Builders Ecole d’Ingénieurs, COMUE Normandie Université, 1 Rue Pierre et Marie Curie, 14610 Epron, France; hassan.fardoun@outlook.com; 2 RISCO, Department of Civil Engineering, University of Aveiro, 3810-193 Aveiro, Portugal; victorf@ua.pt; 3 CICECO, Department of Materials and Ceramics Engineering, University of Aveiro, 3810-193 Aveiro, Portugal; mantas@ua.pt

**Keywords:** thermoactivated recycled cement, hardened cement powder, hydrated lime, strength development

## Abstract

Thermoactivated recycled cement (RC) is a growing area of research and development in the cement industry. The approach represents a reversible process of cement hydration in which dehydrated compounds with similar characteristics to cement are obtained by means of thermal activation. To avoid CO_2_ emissions during the production of such RC, this study assesses the possibility of replacing ordinary Portland cement (OPC) with hardened cement powder (HCP) prepared with different proportions of hydrated lime (HL), relying on a second pozzolanic reaction, and compares it with RC mortars. Due to the thermal activation of HCP, the compressive strength increases by 11.5%. The addition of 8% HL produced an important increase in strength from 28 days to 90 days by 12.8%, although without surpassing the strength values of mortar produced only with HCP or with RC. The compressive strength results suggest the existence of a secondary pozzolanic reaction when using HCP from a cement paste source, but such a pozzolanic reaction was fully perceived in XRD patterns when using concrete as parent material, unlike cement paste, possibly due to large crystalline sand peaks that could have hindered the effective identification of smaller crystalline peaks.

## 1. Introduction

Approximately 10 billion tons of construction and demolition waste (CDW) are generated globally each year [1]. CDW valorization as recycled aggregate in concrete has been widely investigated. It is reported that recycled aggregate concrete demonstrates lower mechanical and durability properties than ordinary concrete due to the cement mortar present in the aggregates surface [2]. Subsequently, different approaches have been proposed to remove the attached mortar off the recycled aggregate, such as heat treatment, ultrasonic cleaning and pre-soaking in acid. Other enhancement methods can include surface coating on recycled aggregate (pozzolanic materials, polymer emulsion) and CaCO_3_ precipitation in order to improve the performance of recycled aggregate concrete [3,4]. 

Although hardened cement paste accounts for a considerable amount of CDW, there is a relatively low number of studies on recycled cement (RC) compared to recycled aggregate [5], and more attention is required on the former. Indeed, cement is the main contributing material to the carbon footprint of concrete. It is estimated that approximately 0.8 tons of CO_2_ are emitted for each ton of cement produced [6]. Cement manufacturing accounts for more than 80% of CO_2_ emissions of concrete production [7], representing around 7% of the global anthropogenic CO_2_ emissions [8,9]. 

In recent years, studies have been frequently published on thermoactivated recycled cement [10,11,12,13,14,15,16,17,18]. The process involves crushing and sieving old cementitious source material to obtain hardened cement powder (HCP). The latter is then dehydrated by means of thermal activation, followed by a cooling phase. Old cement paste is mainly adopted by researchers as a source material rather than concrete or mortar to avoid undergoing the separation process of hardened cement paste from coarse and fine aggregate [18]. However, Bogas et al. [11] replaced ordinary Portland cement (OPC) with thermoactivated RC generated from old cement paste and old concrete. At 50 wt.% replacement ratio, the 28 days-compressive strength of mortars was reported to be 17.9 MPa and 13.8 MPa, respectively. The heating temperature is a crucial parameter that highly affects the properties of thermoactivated RC. Wang et al. [19] investigated the effect of different heating temperatures (120 °C, 450 °C, 750 °C and 1150 °C) on the compressive strength of RC paste. The highest compressive strength of RC paste was reported at 450 °C. In another study [17], a heating temperature of 650 °C yielded RC pastes of higher compressive strength than pastes produced with RC previously thermally activated at 450 °C and 850 °C. Therefore, the optimal dehydration temperature may vary according to the characteristics of the parent material, and thermogravimetric analysis is required to select the best heating temperature, as will be discussed later in this study. It is worth noting that a heating rate of 1–20 °C/min is generally considered to attain the desired heating temperature. In addition, the heating dwell time mostly adopted ranges between 2 and 3 h [18]. Thermoactivated RC is usually cooled naturally to reach the room temperature with no evidence be available on the effectiveness of rapid cooling [20]. 

HCP can be considered recycled concrete fines. While the latter corresponds to the fine fractions of waste concrete generated during the recycling process, the former is supposed to be the cement powder of waste concrete. Recycled concrete fines is an emerging supplementary cementitious material (SCM) and HCP is alleged to be so. SCMs have been increasingly replacing OPC. On the other hand, hydrated lime (HL) is frequently adopted as an activator of these by-products as it increases the concentration of Ca(OH)_2_ and facilitates the pozzolanic reaction. Valcuende et al. [21] reported 29% increase in compressive strength after 10% addition of hydrated lime to concrete mixtures with 50% of fly ash. This is attributed to the reaction between Ca(OH)_2_ from hydrated lime and SiO_2_ from fly ash that subsequently generated C–S–H. Zhang et al. [22] assessed the effect of 1%, 2%, 3%, 5% and 7% addition of hydrated lime (by mass of binder) on ultra-high performance concrete containing cement and different SCMs. The highest compressive strength was reported at 5% addition, which reflected an optimal balance between the pozzolanic reaction and cement hydration which generates Ca(OH)_2_ (followed by the 7% addition). Meanwhile, Yang et al. [23] reported 32.9% and 31.66% of SiO_2_ and CaO in recycled cement powder obtained from cement paste.

HCP from CDW is assessed in thermoactivated RC to avoid landfill and reduce the consumption of raw materials required in cement production and, most importantly, to reduce CO_2_ emissions. Manufacturing thermoactivated RC generates much less CO_2_ than OPC production. Indeed, while the thermal activation temperature of RC is 600 °C (±100 °C), the production temperature of OPC is around 1450 °C (±100 °C). Wang et al. [17] stated that 70.5–78.4% CO_2_ emissions and 35–61.5% energy consumption could be reduced in RC production compared to OPC production. This study assesses the mechanical properties of mortars containing OPC, RC and HCP with different portions of hydrated. It aimed to select the optimal proportion of hydrated lime. A better mechanical performance of the mixture with hydrated lime can eliminate the need for thermal activation and thus the production of thermoactivated RC. 

## 2. Experimental Procedure

### 2.1. Concrete as a Binder Source

**Figure 1:**
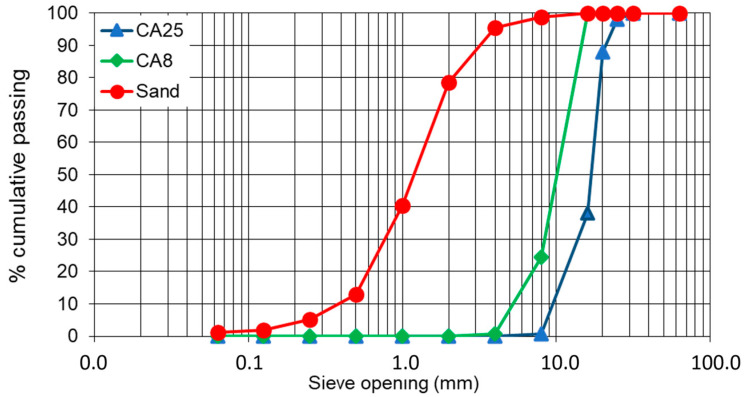
Particle size distribution of sand and coarse aggregates (CA8 and CA25).

Concrete samples were prepared and considered as a binder source material for this research work. The concrete samples included 300 kg/m^3^ of CEM II/B-L 32.5 N (Cimpor, Souselas, Portugal) and possessed a *w*/*c* of 0.66. Coarse aggregates of maximum size 25 mm (named as CA25) and 8 mm (CA8) have been used, along with coarse sand of fineness modulus 3.68 which were used for casting the precursor material. Figure 1 shows the particle size distribution of the sand and coarse aggregates. The samples were demolded 24 h after casting and transferred to a curing room with controlled conditions at 20 ± 2 °C and >95% relative humidity (RH). The average 28 days-compressive strength of these concrete samples was 18 MPa. 

### 2.2. Separation Process to Obtain HCP

**Figure 2:**
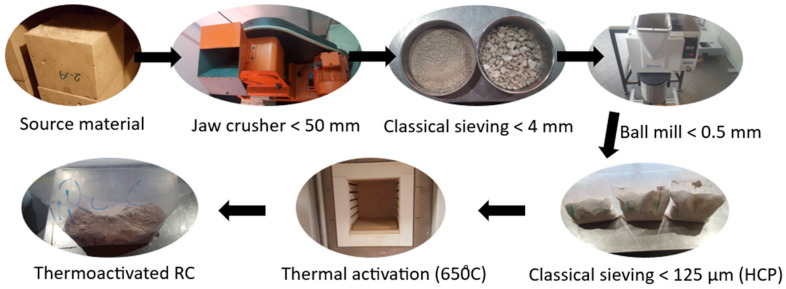
Production of HCP and thermoactivated RC.

After two months of curing, the source concrete cubes were taken out of the controlled curing chamber to separate HCP from other components. Separation was carried out in four main stages. In the first stage, a jaw crusher transformed concrete into particles of 50 mm maximum size. In the second stage, classical laboratory sieving was carried out and particles above 4 mm were removed. This stage eliminated the coarse aggregate. Attempts were made to remove the attached mortar on recycled coarse aggregate by a manual mortar pestle, and the remaining material was added to the material still under separation. In the third stage, a ball mill was used to transform the material into powder that passes through a sieve opening of 0.5 mm. In the fourth and final stage, classical laboratory sieving was carried out once again and particles less than 0.125 mm (125 µm) were considered as HCP. This final stage aimed to eliminate as much as possible the sand particles above 125 µm. Figure 2 illustrates the steps followed to produce HCP (and thermoactivated RC). 

### 2.3. Thermal Activation to Obtain Thermoactivated RC

**Figure 3:**
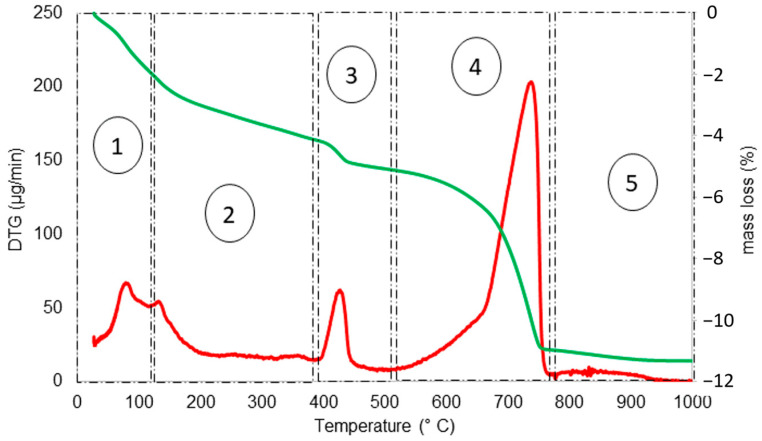
Water loss (%—green line) and DTG (µg/min—red line) of HCP sample (from concrete source) as function of temperature. Phase 1: Evaporation of free water; Phase 2: Decomposition of ettringite and gypsum, and dehydration of C–S–H; Phase 3: Calcium hydroxide dihydroxylation; Phase 4: Decomposition of calcium carbonates, and Phase 5: Decarbonation of well-crystalline calcite.

Figure 3 shows the water loss (%) and DTG (µg/min) of the HCP sample as function of thermal activation temperature. For the HCP in this study, the first peak in DTG was noticed at 85 °C. Up to 105 °C, this stage is related to the evaporation of free water [24]. Stage two of range 105–380 °C and peak 185 °C is associated with the decomposition of ettringite and gypsum, and dehydration of C–S–H [11]. Phase three of range 380–510 °C and peak 430 °C is mainly related to calcium hydroxide (portlandite-CH) dihydroxylation [25,26]. The fourth phase corresponds to temperature range of 510–780 °C with 740 °C DTG peak, and is linked to the decomposition of calcium carbonate (calcite-CaCO_3_) precipitated from C–S–H carbonation and other unstable components. For temperatures above 780 °C, decarbonation of well-crystalline calcite formed by portlandite carbonation takes place [24,27,28]. 

The C-S-H decomposition has been reported to take place over a wide variety of temperatures [18]. It has been reported that slight dehydration of C–S–H gel may begin to occur as early as 100 °C [29]. However, it is known that C–S–H, for temperatures above 800 °C, transforms into wollastonite (CaSiO_3_), which is an unreactive phase [30]. Thus, heating temperatures lower than 800 °C have to be selected. Furthermore, it is beneficial to conserve the calcite in the precursor material so it can serve as an accelerating agent for the hydration of recycled cement [17]. In addition, this minimizes CO_2_ emissions associated with higher firing temperatures. According to Figure 3, the sharp increase in phase 4 starts at the temperature of 662 °C. Conversely, lower temperature yields RC of bigger particle size, while jennite and tobermorite lose less water which leads to less absorbing and bonding of water during hydration, and both adversely contribute to lower strength development [19]. Therefore, a heating temperature near to 662 °C was considered adequate and thermal activation was carried out at 650 °C. 

Thermal activation took place one week before the casting of new mortars. A heating rate of 10 °C/min was considered to attain the 650 °C. In addition, after heating, the RC was kept in the furnace to reach room temperature by natural cooling. This thermoactivated RC was then conserved in sealed bags. 

### 2.4. Mix Design, Casting and Curing

A mortar mixture possessing 1:3:0.5 of OPC, sand and water, respectively, was considered as the reference. The same sand used in the source material was used for preparing the new mortars, taking into consideration the sieving out of any particles of a size not less than 4 mm. In another two mortar formulations, cement (OPC) was replaced with 25% of thermoactivated RC and HCP to form the designated M-RC and M-HCP mortars, respectively. Three additional mortar formulations produced were 2%, 5% and 8% of hydrated lime (% by mass of cement and HCP) as added to M-HCP to form M-HCP2HL, M-HCP5HL and M-HCP8HL mortars, respectively. The amount of water was kept constant in the six mixtures. The amount of superplasticizer used (SP) (% by mass of the binder (cement + RC + HCP + HL)) varied accordingly to maintain a similar workability (spread). Table 1 shows the mix design of these six mixtures.

For the mixtures of two or more binder materials, the latter was mixed for 1 min in an electrical mixer (KitchenAid KSM 150, Benton Harbor, MI, USA). The sand was then added and mixed with the binder for additional 3 min. Water and superplasticizer were then added to the dry components within 10 s, with mixing continued for an additional 3 min following the addition of water.

After casting, mortars vibration took place for 12 s in a vibrating table. The specimens were demolded 24 h later and transferred to a curing room set in controlled conditions of 20 ± 2 °C and >95% RH. 

### 2.5. Characterization Tests

The following tests were performed on the different mortars to evaluate the effect of replacing OPC as a binder. The tests involved fresh and hardened state characteristic evaluation, together with compositional assessment:

- Workability of mortars was evaluated through slump tests in accordance with EN1015-3 [31].
- Mechanical characterization: compressive and flexural mechanical tests were carried out at the age of 7, 28 and 90 days on 4 × 4 × 4 cm^3^ cubes and 4 × 4 × 16 cm^3^ prisms, respectively, according to EN 1015-11 [32]. Three specimens were tested per mixture and curing age.
- Thermogravimetric analysis was conducted as per [33], using a Labsys TG-DSC16 thermal analyser (Setaram, Carcavelos, Portugal).
- X-ray diffraction (XRD) mineralogic composition analysis: an assessment of HCP was carried out using XRD. The data were collected using X’Pert-Pro MPD Philips/Panalytical diffractometer (Malvern Panalytical Ltd, Worcestershire, United Kingdom) equipped with K Cu radiation (λ = 1.5405 Å) operating at 40 mA and 45 KV. The scanning was conducted (2θ, Cu-kα) between 5° and 60° at a scan speed of 0.02°/s [34].

Capillary absorption: water absorption tests were carried out after 90 days of drying. The bottom side of each specimen was submerged in 2 mm of water. The mass of the specimens was measured periodically [35]. The list of characterization tests performed and standards followed is summarized in Table 2.

## 3. Results and Discussion

### 3.1. Workability

Formulations of tested mixtures were designed in order to avoid any significant workability loss. Hence, the amount of water was kept constant in all mixtures while SP was varied to adjust workability (230 ± 10 mm). According to Table 1, it can be stated that RC, HCP and HL may require additional water or SP to maintain flowability. The higher porosity of RC and HCP can justify their higher water demand. In addition, the generation of free lime (CaO) and the increase in the surface area on dehydrated phases can contribute to increase absorption capacity of RC [36], as well as the water entrapped in the flaky layers of RC particles [37]. 

### 3.2. Compressive and Flexural Strength Strength

**Figure 4:**
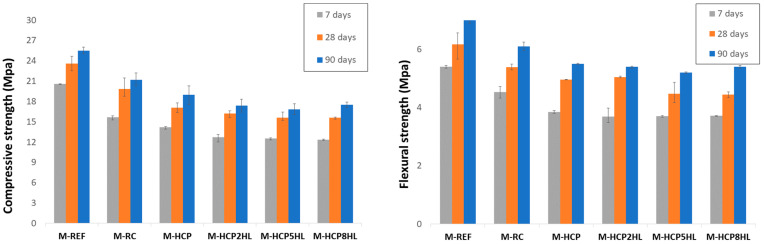
Compressive and flexural strengths results at different curing ages.

Figure 4 shows the compressive and flexural mechanical strength of all mixtures at different curing ages (7, 28 and 90 days). Strength increases as expected with age due to cement hydration, and decreases as OPC content is reduced. Although this reduction is not significant, interesting mechanical strength values are still achieved in the formulated samples. The compressive strength of REF samples revealed, of course, the highest strength, followed by M-RC, which in turn indicate lower cementitious properties of RC than OPC. The replacement of OPC by 25% of RC reported a 31.1%, 18.8% and 20.2% loss in compressive strength at 7, 28 and 90 days, respectively. The decrease in such percentages compared to 7 days may be due to more formation hydration products (particularly C-S-H) at advanced curing age, which can fill pores and thus reduce the overall porosity. On the other hand, the thermal activation of HCP yielded a strength increase by 11.5% at 90 days. The higher strength of M-RC compared to M-HCP may also point to an effective rehydration of dehydrated products of RC obtained due to thermal activation, in addition to a higher porosity existing in the latter. The mixtures incorporating hydrated lime showed slightly lower strength than M-HCP. M-HCP8HL, with slightly higher strength than other mixtures with hydrated lime, demonstrated a compressive strength of 17.5 MPa at 90 days, less than 8.57% M-HCP. Despite such strength loss, the increase of 6.9%, 7.94% and 12.18% in compressive strength and of 7.1%, 16.33% and 21.62% in flexural strength for M-HCP2HL, M-HCP5HL and M-HCP8HL from 28 days to 90 days, respectively, might indicate that a secondary pozzolanic reaction could have taken place with HL proportion of 8%, which could be an optimal proportion compared to 2% and 5%.

### 3.3. Mineralogic Characterization by XRD

**Figure 5:**
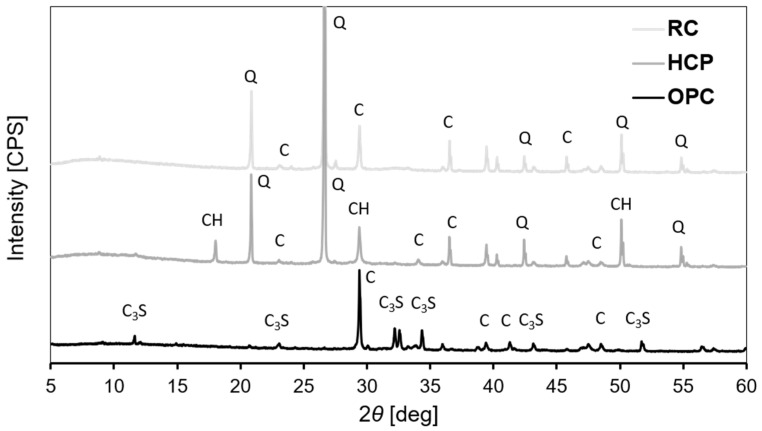
XRD of OPC, HCP and RC (Q: Quartz; C: CaCO_3_; CH: calcium hydroxide; C_3_S: Tricalcium silicate).

X-ray diffraction is a non-destructive testing method for determining a range of mineralogical characteristics of materials, including the type and quantities of phases present. Figure 5 shows the XRD spectra of OPC, HCP and RC (powders used to produce mortars). It is possible to observe that peaks of quartz are present in HCP and RC. The presence of quartz can be related to traces of sand, although a separation process took place. Such crystalline peaks indicate that HCP does not possess a mainly amorphous structure. 

It appears also that calcium silicate or dehydrated products are not present in RC. In fact, the XRD spectrum of OPC shows tricalcium silicate (C_3_S) with no dicalcium silicate (C_2_S). Researchers have found that new recycled cement does not possess C_3_S or that C_3_S cannot be formed at 650 °C but only C_2_S [11]. As long as C_2_S is not present in OPC, how could it be formed in RC? This is to be verified later in this article. Accordingly, these results indicate that the higher strength of M-RC compared to M-HCP cannot be attributed to dehydrated products as they were not generated due to thermal activation according to XRD analysis. The higher strength of M-RC can be attributed to the decrease in particle size due to thermal treatment. 

**Figure 6:**
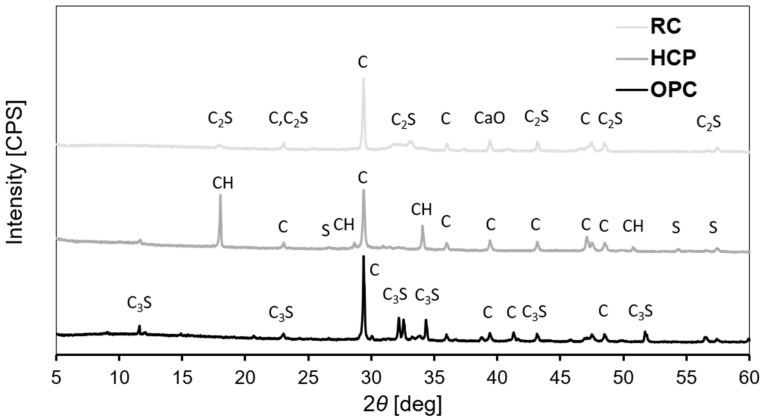
XRD of HCP and RC from cement paste source in addition to OPC (S: SiO_2_; C: CaCO3; CH: calcium hydroxide; C_2_S: dicalcium silicate, C_3_S: Tricalcium silicate, CaO: free lime).

Figure 6 shows the XRD spectra of HCP and RC that were obtained from cement paste (casted, cured and crushed similarly as the concrete source material (see Section 2.1 and Section 2.2)) in addition to OPC. Silicon dioxide (SiO_2_) is present in HCP from cement paste source, unlike powders from concrete source. Moreover, dicalcium silicate (C_2_S) is present in RC from cement paste source, unlike powders from concrete source in addition to CaO. 

Thus, C_2_S was formed in RC from cement paste, though OPC did not show this chemical compound by XRD. This indicates that C_2_S is present in this type of cement but was not captured during our laboratory test. Indeed, by looking at past studies that carried out XRD analysis on CEM II/B-L 32.5 N ([24]), XRD detected minor levels of C_2_S in this type of cement. Knowing that SiO_2_ and C_2_S are present in minor quantities, if they exist, in HCP and RC of concrete source (respectively), this may justify the incapability to identify them in this study by XRD analysis. The large peaks of quartz could have contributed to the masking of such smaller peaks. 

**Figure 7:**
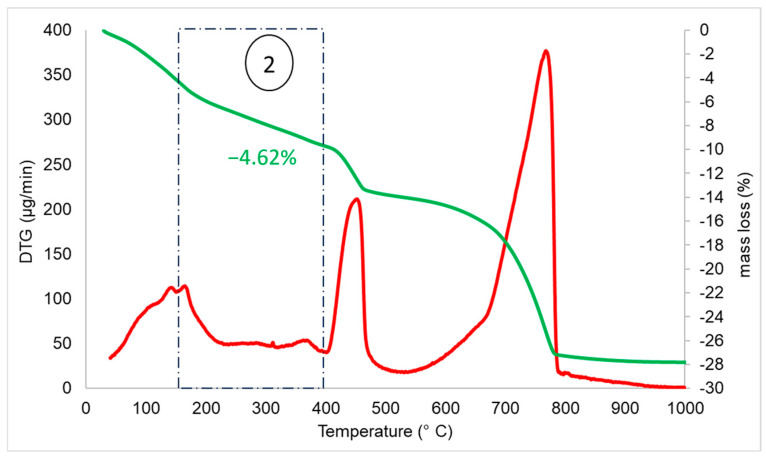
Water loss (%—green line) and DTG (µg/min—red line) of HCP sample (from cement paste source) as function of temperature. Phase 2: Decomposition of ettringite and gypsum, and dehydration of C–S–H.

Furthermore, according to Figure 3, the weight loss during phase two, which is generally correlated to the dehydration of C–S–H, is not more than 1.85% and thus dehydration products are not significant. Figure 7 shows the thermogravimetric analysis of HCP from cement paste. During phase 2, a mass loss of 4.62% is reported (Figure 7). 

These are possible assumptions. Nevertheless, it could be also that neither SiO_2_ is present in HCP nor C_2_S in RC from concrete source. More experimental investigations are required.

### 3.4. Capillary Water Absorption

**Figure 8:**
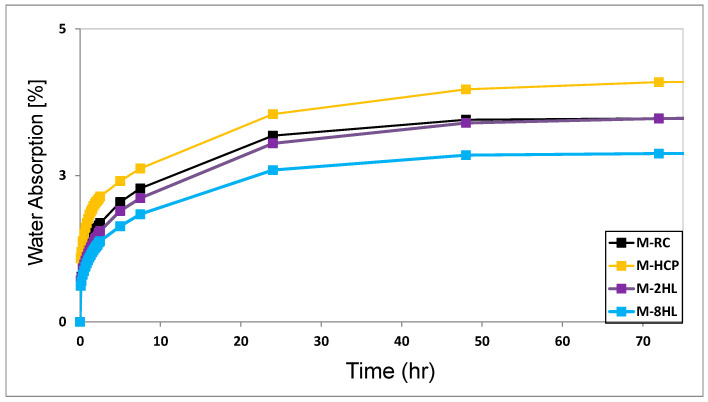
Capillary rise of M-RC, M-HCP, M-2HL and M-8HL.

Figure 8 shows the capillary water absorption results of M-RC, M-HCP, M-2HL and M-8HL samples. The four mixtures follow the same absorption profile. First, a sharp water absorption took place during the first two hours. Absorption continues at a lower rate even after 48 h. Saturation seems to be attained around 72 h in all mixtures but to a lower extent in M-HCP. At 72 h, the water absorption reported 4.11% for M-HCP. Such a value decreased by almost 18% due to thermal activation. The higher water uptake of M-HCP than M-RC indicates a higher and finer porosity in the former. 

In contrast, the addition of 2% and 8% of HL decreased the water absorption of M-HCP by 14% and 42%, respectively. As no evidence of additional binging phases being formed by lime addition has been provided by XRD analysis, the decrease in water absorption may indicate that HL has mainly a filler effect [38]. However, such effect did not improve the compressive strength. Here, it is worth noting that various fillers are alleged to improve durability rather than the mechanical properties [39], which may be due to a densification effect delivered by the filler [40]. 

## 4. Conclusions

OPC in the reference mortar (REF) was partially (25%) replaced with RC (M-RC), HCP (M-HCP) and HCP with 2%, 5% and 8% of hydrated lime. Compressive and flexural tests were carried out at different curing ages. The following can be stated:

- The replacement of OPC by 25% of RC reported a 31.1%, 18.8% and 20.2% loss in compressive strength at 7, 28 and 90 days, respectively.
- The thermal activation at 650 ° C of HCP reported an increase in compressive strength by 11.5%.
- Though XRD carried on at HCP from concrete source did not show the presence of SiO_2_, M-HCP8HL reported 12.18% increase in compressive strength and 21.62% in flexural strength from 28 to 90 days more than any other mixture. Further experimental investigations are required to examine HCP from cement paste as the XRD conducted on the latter demonstrated the presence of SiO_2._

## Figures and Tables

**Table 1:** Mix design of all mixtures.

	Cement (g)	RC (g)	HCP (g)	HL (g)	*w*/*c*	Sand (g)	SP (%)	Slump (mm)
REF	500	-	-	-	0.5	1500	0.2	230
M-RC	375	125	-	-	0.5	1500	0.3	225
M-HCP	375	-	125	-	0.5	1500	0.2	220
M-HCP2HL	375	-	125	10	0.5	1500	0.2	240
M-HCP5HL	375	-	125	25	0.5	1500	0.25	235
M-HCP8HL	375	-	125	41	0.5	1500	0.27	225

**Table 2:** Tests conducted in this study.

Fresh Properties	Hardened Properties		Microstructural Assessment		Durability
Workability	Compressive Strength	Flexural Strength	Thermogravimetric Analysis	X-ray Diffraction	Capillary Absorption
EN 1015-3 [31]	EN 1015-11 [32]		C1872-18 [33]	EN 13925-1 [34]	EN 1015-18 [35]

## Data Availability

Data available on request.
